# Aberrant salience in cannabis-induced psychosis: a comparative study

**DOI:** 10.3389/fpsyt.2023.1343884

**Published:** 2024-01-08

**Authors:** Valerio Ricci, Ilenia Di Muzio, Franca Ceci, Francesco Di Carlo, Gianluca Mancusi, Tommaso Piro, Andrea Paggi, Mauro Pettorruso, Federica Vellante, Domenico De Berardis, Giovanni Martinotti, Giuseppe Maina

**Affiliations:** ^1^Department of Neuroscience, San Luigi Gonzaga University Hospital, Orbassano, Italy; ^2^Department of Neurosciences, Imaging and Clinical Sciences, Università degli Studi G. D’Annunzio Chieti-Pescara, Chieti, Italy; ^3^Department of Mental Health, ASL Lanciano-Vasto-Chieti, Chieti, Italy; ^4^NHS, Department of Mental Health, Psychiatric Service for Diagnosis and Treatment, Hospital “G. Mazzini”, Teramo, Italy; ^5^Department of Clinical and Pharmaceutical Sciences, University of Hertfordshire, Hatfield, United Kingdom

**Keywords:** natural cannabis, synthetic cannabinoids, psychosis, spice, aberrant salience, substance use disorder, substance-induced psychosis

## Abstract

**Background:**

Natural Cannabis (NC) and Synthetic Cannabinoids (SCs) use can increase the risk and exacerbate the course of psychotic disorders. These could be influenced by the Aberrant Salience (AS) construct. It refers to an excess of attribution of meaning to stimuli that are otherwise regarded as neutral, thereby transform them into adverse, dangerous, or mysterious entities. This leads the patient to engage in aberrant and consequently incorrect interpretative efforts concerning the normal perception of reality and its relationship with our analytical abilities. AS appears to play a significant role in the onset and perpetuation of psychotic disorders. The internal conflict arising from aberrant attributions of significance leads to delusional thoughts, ultimately culminating in the establishment of a self-sustaining psychosis.

**Aims:**

To examine the differences between psychoses course not associated with cannabis use and those associated with NC-use and SCs-use, in terms of psychotic and dissociative symptoms, AS, global functioning and suicidal ideation.

**Methods:**

A sample of 62 patients with First Episode Psychosis (FEP) was divided into 3 groups: non cannabis users (non-users, *N* = 20); NC-users or rather Delta-9-tetrahydrocannabinol (THC) users (THC-users, *N* = 21); SCs-users, commonly referred to as SPICE-users (SPICE-users, *N* = 20). Each group underwent assessments at the onset of psychotic symptoms, as well as at the 3 months and 6 months marks, utilizing a range of psychopathological scales. These included the Positive and Negative Syndrome Scale (PANSS) for investigating psychotic symptoms, the Global Assessment of Functioning (GAF) scale for assessing overall functioning, the Dissociative Experiences Scale (DES-II) for measuring dissociative symptoms, the Scale for Suicide Ideation (SSI) for evaluating suicidal ideation and the Aberrant Salience Inventory (ASI) scale for gauging AS.

**Results:**

SPICE-users showed more severe and persistent positive symptoms, while negative symptoms were mostly represented among non-users. Non-users showed better recovery than SPICE-users in global functioning. All groups showed a decrease in both ASI scores and subscale scores. SPICE-users exhibited higher global AS scores and less improvement in this aspect compared to other groups.

**Conclusion:**

This study may help understanding the role of AS in both non-substance-related and substance-induced psychosis. This knowledge may lead clinician to a better diagnosis and identify patient-tailored psychopharmacological treatment.

## 1 Introduction

### 1.1 The involvement of natural cannabis and synthetic cannabinoids in the emerging landscape of novel psychoactive substances

Cannabis is a widely consumed recreational psychoactive substance, with approximately 192 million users globally in 2018, accounting for about 3.9% of the world’s population aged 15 to 64 (1). According to the European Monitoring Centre of Drugs and Drug Addiction (EMCDDA), Italians who in 2017 declared having used cannabis at least once in their life represented 32.7% of the population; the predominant consumer group (20.9%) comprises young adults aged 15 to 34 (2).

The popularity of cannabis is attributed to its affordability, accessibility, and distinct psychotropic effects, including feeling euphoria and relaxation. However, about one in ten users develop problematic usage patterns (3) leading to a medical condition known as Cannabis Use Disorder (CUD) as defined by DSM-5 (4). The primary psychoactive compound in cannabis is Δ9-tetrahydrocannabinol (THC), which acts as a partial agonist on cannabinoid receptors CB1 and CB2.

In addition to Natural Cannabis (NC), there are Synthetic Cannabinoids (SCs), often called “Spices,” which produce more potent psychotropic effects than NC. SCs have become the most prevalent category of New Psychoactive Substances (NPS) in use (5). NPS represents a new challenge in the fight against illicit drugs: they are advertised as “legal highs,” suggesting that, in contrast to substances regulated by the national laws, trading with NPS is legal. At the end of 2019, the EMCDDA was monitoring around 790 NPS, 53 of which had been reported for the first time in Europe in 2019, a number which represents a decrease compared with data previously recorded, reflecting the results of sustained efforts to restrict NPS production and control their diffusion. NPS are not detectable in common practice examinations, yet they sustain relevant psychotropic effects similar to common illicit substances. NPS and SCs in particular, constitute a significant health issue due of their widespread availability, easily found and purchased online (6) and in convenience stores (7, 8). SCs primarily induce their psychoactive effects by binding strongly to CB1 receptors, sometimes acting as full agonists, also leading to heightened toxic symptoms like anxiety, paranoia, tachycardia, and hypertension (9). Regarding this last point, recently, SCs have been showed to possess I-MAO properties. In particular, a pressor response to tyramine, accelerated by smoking SCs, could provide an explanation for the severe and unpredictable hypertensive side effects recorded in SCs-users (10).

The mechanisms responsible for the potential of both NC and SCs to trigger psychotic symptoms, while not fully understood, seem to involve interactions with dopamine, gamma-aminobutyric acid (GABA), and glutamatergic systems. Firstly, CB1 and dopaminergic receptors (D2) are co-expressed in mesolimbic system, where cannabis may increase dopaminergic activity through intracytoplasmic and epigenetic pathways, thus contributing to the development of positive symptoms (11). Furthermore, CB1 receptors modulate the GABAergic system by reducing GABA release in hippocampus and prefrontal cortex, inducing a disinhibition of pyramidal cell activity, and consequently, psychotomimetic effects. Lastly, in brain regions of glutamatergic system (hippocampus, prefrontal cortex, amygdala) NC and SCs can reduce glutamate release by a N-methyl-D-aspartate (NMDA) receptor disruption, possibly inducing psychosis (12, 13).

### 1.2 Psychosis and use of cannabis

Psychosis is characterized by a disturbance in an individual’s ability to accurately assess and comprehend reality, including symptoms such as hallucinations, delusions, disorganized thinking and behavior, and negative symptoms like abulia, alogia, anhedonia, and asociality (4). A First Episode Psychosis (FEP) is the initial occurrence of positive psychotic symptoms, such as delusions, hallucinations, or significant disorganized behavior in a patient (14).

Scientific literature strongly supports a robust association between cannabis use and psychosis (15–17), particularly in individuals with a genetic vulnerability, increasing the risk of early-onset psychosis and worsening the severity of symptoms (18, 19). Specifically, cannabis use is strongly linked to the onset of FEP (20) carrying a risk that is two times higher (15, 17) to four time higher (16) for experiencing a psychotic disorder than non-users (21). Furthermore, recent observations suggest that the early onset of cannabis consumption significantly increases the risk of developing psychotic symptoms, especially when initiated before the age of 15 and continuing into adulthood (22). This risk further increases if the consumption involves SCs (23). This new, acute, and more severe presentation of psychosis has been recently termed “Spiceophrenia” (24).

In recent years, numerous studies have been dedicated to characterizing and discerning, at the psychopathological level, psychotic disorders that are unrelated to substance use from those specifically associated with the use of NC and SCs.

The available literature suggests that individuals who use cannabis tend to experience more severe and enduring positive symptoms compared to those who did not use NC and/or SCs. Additionally, these individuals may exhibit less pronounced negative symptoms (21, 25–27). Cannabis-related psychosis is associated with an increased number of hospitalization days (28), lower therapy response (29), decreased pharmacological compliance (30), and a higher risk of relapse (31). Specifically, users of SCs face a heightened risk of experiencing acute psychosis marked by more pronounced positive symptoms and fewer negative symptoms (23, 32, 33) as well as enduring psychotic disorders and relapses (24, 34–38). Regarding overall functioning, individuals experiencing psychosis associated with the use of NC and SCs tend to exhibit poorer global functioning compared to those with psychosis who have never used cannabis (27, 39).

A symptom that has received relatively less attention but appears to play a significant role in psychosis is dissociation (40). Dissociation often presents as a disturbance in self-perception, sensory experiences, derealization, and depersonalization, potentially leading to the development of self-harming behaviors, increased aggressiveness, and difficulties in regulating emotions. This contributes to higher rates of suicidal ideation and suicide attempts among individuals with cannabis-related psychosis (41). Recent studies have indicated that psychoses linked to NC and/or SCs use are associated with more prominent dissociative symptoms, with a greater tendency for these symptoms to persist over time, particularly in NC-users (21, 42). While there is no direct link between dissociation and suicide (21, 42), there is an increased incidence of suicidal thoughts among SCs-users, potentially driven by SCs-induced impulsivity and the emergence of positive symptoms (42).

### 1.3 Aberrant salience, cannabis, and psychosis

The concept of ‘Aberrant Salience’ (AS) is closely associated with the psychopathology of psychosis and involves attributing inappropriate significance to typically irrelevant stimuli (43). This cognitive process is linked to dysregulated dopaminergic neurons and plays a crucial role in the development and perpetuation of psychotic disorders by generating delusional thoughts (44). Recent research has indicated that cannabis users often exhibit elevated levels of AS, which is associated with the presence of positive psychotic symptoms. AS appears to be closely connected to cannabis use, suggesting a potential relationship between AS and the effects of cannabis on psychosis (45). In this context, AS may act as a predisposing pre-clinical risk factor for the development of psychotic disorders among cannabis users, but further research is needed to fully understand this complex relationship (46). To assess AS Cicero et al. (47) introduced the Aberrant Salience Inventory (ASI) scale, a self-reported questionnaire. Elevated scores on ASI scale have been linked with psychosis proneness, which includes schizotypy or schizotypal traits (47). The ASI is scored based on five correlated subscales: Feelings of Increased Significance (FIS) heightened salience to otherwise innocuous stimuli; Sense Sharpening (SS), anomalies of perception; Impending Understanding (IU), (heightened salience leading to a breakthrough in understanding); Heightened Emotionality (HE) and Heightened Cognition (HC) (the efforts to comprehend emotions and thoughts accompanying AS experiences, which may also relate to pre-psychotic experiences). Observations have indeed revealed a positive correlation between the number of years of cannabis use and the frequency of use with ASI scores, supporting the growing idea that cannabis use is associated with a higher occurrence of psychotic experiences (48).

The primary objective of our study was to explore the distinctions between psychoses not associated with cannabis use and those linked to the consumption of NC and SCs in terms of: (a) Positive and Negative Syndrome Scale (PANSS) for investigating psychotic symptoms, (b) the Global Assessment of Functioning (GAF) scale for assessing overall functioning, (c) the Dissociative Experiences Scale (DES-II) for measuring dissociative symptoms, (d) the Scale for Suicide Ideation (SSI) for evaluating suicidal ideation, and (e) ASI scale for gauging AS. Additionally, recognizing that AS is a well-established concept implicated in the origin and progression of non-cannabis-induced psychosis, we aimed to investigate whether AS also plays a role in the onset and course of NC and SCs-induced psychosis. Given the lack of robust literature supporting this concept, we set out to examine the relationships between AS and PANSS, AS and GAF, AS and DES II, as well as AS and SSI.

## 2 Materials and methods

### 2.1 Participants

The study included participants who had experienced FEP and were recruited during the acute phase of their psychosis from hospital emergency rooms in the Italian regions of Val d’Aosta and Piemonte, spanning from 2013 to 2022.

Inclusion criteria:

1. Age between 16 and 50 years.
2. Diagnosis of schizophrenia spectrum disorder or another non-affective psychotic disorder based on DSM-5 criteria (4).
3. Individuals with a diagnosis of CUD needed a history of frequent cannabis use (2–3 times per week).
4. Individuals without a diagnosis of NC/SCs use disorder were required to have no lifetime use of NC or SCs.
5. Primary residence within the recruiting area.

Exclusion criteria:

1. Individuals who had previously sought mental health services for psychosis.
2. Participants who had received prior treatment with antipsychotic medications.
3. Individuals with intellectual disability (QI score less than 70).
4. Any lifetime history of significant medical illness.
5. Participants reporting the use of substances other than cannabis at a frequency greater than once a month.
6. Individuals with an alcohol use disorder.

After explaining the study thoroughly, 62 participants gave written informed consent and were recruited over 9 years. They were divided into three groups with similar numbers and age and sex distributions. However, during the follow-up, 25 patients, around 3 per year, dropped out of the study, resulting in a dropout rate of about 28.7%. This dropout rate is crucial to consider when analyzing and interpreting the study’s findings.

In the study, all patients received antipsychotic treatments tailored to their medical history and condition, following clinical guidelines (49). Prospective evaluations occurred at two time points: after 3 months (T1) and after 6 months (T2). These assessments aimed to gauge the initial effects of treatment and the discontinuation of NC and SCs at T1, as well as the individuals’ conditions after a period of relative stabilization at T2. This timeline allowed researchers to monitor changes and progress over time in response to treatment and abstinence from cannabis use. Substance use was evaluated through a comprehensive assessment of participants’ substance use history, including the Drug Abuse Screening Test-10 (DAST-10), a general tool for assessing drug-related issues (49). The use of NC and SCs was assessed using the Cannabis Use Disorders Identification Test-Revised (CUDIT-R), designed to screen for CUD (50). The specific type of cannabis used by participants was determined through clinical interviews (51) conducted with both patients and their family members. The study sample was divided into three distinct groups based on their cannabis consumption habits, and when applicable, the type of cannabis consumed. These groups likely included:

-Non-users.

-THC-users.

-SPICE-users.

### 2.2 Measurements

The study utilized several psychopathological scales administered to participants at three different time points, T0, T1 and T2:

-The PANSS (52), a 30-item questionnaire, divided into three subscales, measuring positive and negative symptoms and the general severity of illness.

-The GAF scale (53), a clinician-rated scale measuring psychological, social, and occupational functioning on a continuum from 0 to 100 (25, 27, 54, 55).

-The DES II (56), a self-report questionnaire measuring dissociative experiences, such as derealization, depersonalization, absorption, and amnesia (57). The DES (58) comprises 28 items based on the assumption of a ‘dissociative continuum’ ranging from a mild alteration to severe dissociation.

-The SSI (59), a 19-item scale measuring the intensity, pervasiveness, and characteristics of suicidal ideation in adults, as well as assessing the risk of later possible suicide attempts.

-The ASI scale (47) a 29-item self-report designed to assess the process of giving unusual or incorrect assignment of salience, significance, or importance to otherwise innocuous or irrelevant stimuli. The ASI includes five correlated subscales: (a) FIS, (b) SS, (c) IU, (d) HE, and (e) HC. Scores are calculated by summing the “yes” replies, and a total score is derived from the sum of all items (see Supplementary Material).

### 2.3 Statistical analysis

Shapiro–Wilk test was used to determine whether the data were normally distributed. Comparisons were conducted using ANOVA test with Tukey post-hoc, Chi-square test and Fisher’s exact test, as appropriate. ANOVA test was used for continuous variables, whereas Chi-square test and Fisher’s exact test was used for categorical variables. Correlations were calculated using Spearman’s rho coefficient. Quantitative parameters were presented as mean ± standard deviation (SD) and the qualitative parameters as number and percentage per class. The significance level was set at *p* < 0.05. The analysis was performed using IBM SPSS for Windows version 22.

### 2.4 Ethics statement

All participants provided written informed consent after receiving explanations of the study. The study was conducted according to the principles outlined in the Declaration of Helsinki. The study was approved by the SS. Annunziata Hospital – University G. d’Annunzio Ethical Committee (reference code: CHPN189, 26 January 2012).

## 3 Results

### 3.1 Demographics and cannabis use

Sixty-two (60) subjects (male = 31; 50%) with acute psychosis have been included in the analysis, with a mean age of 23.97 years (SD ± 4.58). All subjects have been arranged in three homogeneous subgroups based on cannabis use: non-users (*n* = 20); THC-users (*n* = 22), and SPICE-users (*n* = 20). The three groups were comparable for sex (*p* = 0.466) and age (*p* = 0.080). Regarding pharmacological treatment, in the non-users sample, 11 patients were treated with partial agonists (cariprazine or aripiprazole), 5 with risperidone or lurasidone or paliperidone (−dones), 2 with olanzapine or quetiapine (−pines), and 2 with 1st generation antipsychotics (haloperidol). In the THC-users sample, 10 patients were treated with partial agonists, 6 with -dones, 2 with -pines, and 4 with haloperidol. In the SPICE-users sample, 10 patients were treated with partial agonists, 4 with -dones, 2 with -pines, and 4 with haloperidol (see Table 1).

**Table 1:** Descriptive characteristics of the sample.

	Non-users (*n* = 20)	THC-users (*n* = 22)	SPICE-users (*n* = 20)	Statistics (F/Chi)	*p*
Demographics and cannabis use					
Sex, M	10 (50)	13 (59)	8 (40)	1.527	0.466
Age, years	25.1 ± 4.3	24.6 ± 5.2	22.1 ± 3.7	2.643	0.080
Marital status, single	15 (75)	18 (82)	18 (90)		0.462
Age of onset of cannabis use, years	–	18.5 ± 2.8	19.5 ± 2.7	1.548	0.221
Current psychosis, yes	9 (45)	12 (54)	10 (50)	0.382	0.826
Current cannabis use, yes	6 (30)	8 (36)	5 (25)	4.715	0.318
Comorbid substance use					
Cocaine use, yes	5 (25)	6 (27)	6 (30)	0.126	0.939
Heroin use, yes	4 (20)	7 (32)	1 (5)		0.089
3,4-metilenediossimetanfetamina (MDMA) use, yes	1 (5)	5 (23)	5 (25)		0.190
Ketamine use, yes	3 (15)	4 (18)	4 (20)		0.916
Other substances use, yes	7 (35)	12 (54)	8 (40)	1.779	0.411
Anamnestic information					
Previous hospitalization					<0.001
No	0 (0)	7 (32)	0 (0)		
Yes, voluntary	18 (90)	9 (41)	11 (55)		
Yes, forced	2 (10)	6 (27)	9 (45)		
Number of hospitalizations	1 (0–3)	0 (0–3)	1 (0–3)		
Prior suicidal attempts, yes	5 (25)	7 (32)	8 (40)	1.033	0.597
Familiarity for suicide, yes	3 (15)	7 (32)	6 (30)		0.403
Familiarity for psychosis, yes	13 (65)	9 (41)	8 (40)	3.266	0.195
Antipsychotic medication					
Antipsychotic medication					0.979
1st generation (Haloperidol)	2 (10)	4 (18)	4 (20)		
2nd generation olanzapine or quetiapine (−pines)	2(10)	2(9)	2 (10)		
2nd generation risperidone or lurasidone or paliperidone (−dones)	5 (25)	6 (27)	4 (20)		
Partial agonists cariprazine or aripiprazole	11 (55)	10 (45)	10 (50)		

Percentages are reported in brackets.

The majority of subjects recruited for the study had comorbid use of other substances. In the first group, 25% of subjects consumed cocaine, 20% heroin, 5% 3,4-metilenediossimetanfetamina (MDMA), 15% ketamine, and 35% other unspecified substances. Among NC-users, 27% used cocaine, 32% heroin, 23% MDMA, 18% ketamine, and 54% other unspecified substances. Among SCs-users, 30% consumed cocaine, 5% heroin, 25% MDMA, 20% ketamine, and 40% other unspecified substances. Most of subjects were followed by Addiction Services. Detailed data about characteristics of subjects recruited are reported in Table 1.

### 3.2 Psychometric assessment

Psychometric scales were compared between and within groups at T0, T1, and T2. During the follow up, the psychometric assessment remained the same for all patients, irrespective of the pharmacological therapy used. Moreover, patients received psychotherapeutic support, and approximately 40% of them voluntarily discontinued this intervention. Results of psychometric assessment relative to PANSS, GAF, DES-II, and SSI are reported in Table 2; results of psychometric assessment of SA are reported in Table 3.

**Table 2:** Psychometric assessment between groups and within groups at different timepoints.

	Non-users (*n* = 20)	THC-users (*n* = 22)	SPICE-users (*n* = 20)	F/Chi	*p*	Tukey Post-Hoc
Positive and Negative Syndrome Scale (PANSS) – positive						
T0	23.60 (4.41)	24.36 (4.48)	27.50 (3.10)	5.218	0.008	SPICE-users > Non-users; SPICE-users > THC-users
T1	21.45 (4.33)	21.73 (4.50)	26.10 (3.54)	7.982	0.001	SPICE-users > Non-users; SPICE-users > THC-users
T2	18.35 (3.62)	19.55 (4.49)	25.60 (4.02)	18.367	0.000	SPICE-users > Non-users; SPICE-users > THC-users
Diff. T2-T0	−5.25 (4.45)	−4.82 (2.13)	−1.90 (2.02)	7.211	0.002	SPICE-users < Non-users; SPICE-users < THC-users
*p*	<0.001	<0.001	<0.001			
Positive and Negative Syndrome Scale (PANSS) – negative						
T0	20.30 (3.56)	17.45 (2.74)	15.55 (2.16)	13.869	0.000	Non-users > THC-users; Non-users > SPICE-users
T1	19.45 (3.09)	16.50 (2.82)	14.15 (2.68)	17.163	0.000	Non-users > THC-users > SPICE-users
T2	18.45 (3.52)	15.00 (2.73)	12.85 (3.22)	16.048	0.000	Non-users > THC-users; Non-users > SPICE-users
Diff. T2-T0	−1.85 (1.95)	−2.45 (2.32)	−2.70 (2.08)	0.845	0.435	–
*p*	<0.001	<0.001	<0.001			
Positive and Negative Syndrome Scale (PANSS) – general						
T0	52.35 (4.08)	52.41 (3.43)	53.40 (6.57)	0.301	0.742	–
T1	48.45 (3.94)	49.14 (3.81)	50.05 (6.98)	0.499	0.610	–
T2	45.75 (5.15)	45.27 (4.38)	47.10 (7.03)	0.592	0.556	–
Diff. T2-T0	−6.60 (4.21)	−7.14 (3.09)	−6.30 (3.70)	0.281	0.756	–
*p*	<0.001	<0.001	<0.001			
Global Assessment of Functioning (GAF) scale						
T0	49.45 (5.56)	51.27 (4.92)	50.80 (3.21)	0.844	0.435	–
T1	52.90 (5.78)	53.55 (4.47)	53.15 (3.00)	0.108	0.898	–
T2	58.65 (3.27)	57.14 (5.53)	55.30 (3.34)	3.141	0.051	Non users > SPICE-users
Diff. T2-T0	9.20 (5.77)	5.86 (4.29)	4.50 (4.55)	4.901	0.011	Non users > SPICE-users
*p*	<0.001	<0.001	<0.001			
Dissociative Experiences Scale (DES-II)						
T0	24.55 (4.48)	33.55 (8.81)	34.50 (9.76)	9.414	0.000	THC-users > Non-users; SPICE-users > Non-users
T1	20.90 (5.41)	31.86 (9.08)	33.20 (10.01)	12.968	0.000	THC-users > Non-users; SPICE-users > Non-users
T2	18.45 (5.72)	30.95 (9.24)	30.35 (9.38)	14.625	0.000	THC-users > Non-users; SPICE-users > Non-users
Diff. T2-T0	−6.10 (3.23)	−2.59 (3.50)	−4.15 (3.15)	5.920	0.005	THC-users < Non users
*p*	<0.001	0.004	<0.001			
Scale for Suicide Ideation (SSI)						
T0	2.45 (1.47)	3.27 (1.67)	4.95 (2.19)	10.092	0.000	SPICE-users > Non-users; SPICE-users > THC-users
T1	2.30 (1.38)	2.55 (1.01)	4.35 (1.95)	11.476	0.000	SPICE-users > Non-users; SPICE-users > THC-users
T2	2.35 (1.46)	2.41 (1.10)	4.15 (2.06)	8.550	0.001	SPICE-users > Non-users; SPICE-users > THC-users
Diff. T2-T0	−0.10 (1.02)	−0.86 (1.32)	−0.80 (1.20)	2.586	0.084	–
*p*	0.666	0.009	0.008			

Data are reported as mean (SD). Statistics: one-way ANOVA, Tukey post-hoc test and Wilcoxon test.

**Table 3:** Psychometric assessment of salience between groups and within groups at different timepoints.

	Non-users (*n* = 20)	THC-users (*n* = 22)	SPICE-users (*n* = 20)	Statistic (F)	*p*	Tukey Post-Hoc
Aberrant Salience Inventory (ASI) – total						
T0	15.65 (3.63)	16.91 (4.13)	19.40 (3.84)	4.844	0.011	SPICE-users > Non-users
T1	14.40 (3.66)	15.73 (3.82)	18.45 (3.87)	5.960	0.004	SPICE-users > Non-users
T2	12.45 (3.49)	14.68 (4.06)	18.00 (3.70)	10.994	0.000	SPICE-users > Non-users; SPICE-users > THC-users
Diff. T2-T0	−3.20 (2.21)	−2.23 (1.97)	−1.40 (1.85)	3.994	0.024	SPICE-users > Non-users
*p*	<0.001	<0.000	0.006			
ASI – Feeling of Increased Significance (FIS)						
T0	4.00 (0.97)	3.50 (1.14)	4.00 (0.86)	1.759	0.181	–
T1	3.70 (1.08)	3.05 (1.05)	3.85 (1.09)	3.390	0.040	SPICE-users > THC-users
T2	3.25 (1.16)	3.00 (1.11)	3.75 (0.97)	2.567	0.085	–
Diff. T2-T0	−0.75 (0.79)	−0.50 (1.01)	−0.25 (0.55)	1.891	0.160	–
*p*	0.002	0.040	0.059			
ASI – Senses Sharpening (SS)						
T0	2.80 (0.83)	3.77 (0.81)	4.30 (0.86)	16.589	0.000	SPICE-users > Non-users; SPICE-users > THC-users
T1	2.50 (0.89)	3.82 (1.05)	4.20 (0.83)	18.346	0.000	SPICE-users > Non-users; SPICE-users > THC-users
T2	2.20 (0.77)	3.27 (1.08)	4.20 (0.89)	23.289	0.000	SPICE-users > THC-users > Non-users
Diff. T2-T0	−0.60 (0.60)	−0.50 (0.96)	−0.10 (0.55)	2.591	0.083	–
*p*	0.001	0.021	0.414			
ASI – impending Understanding (IU)						
T0	3.00 (0.79)	3.27 (1.03)	3.65 (1.09)	2.210	0.119	–
T1	2.75 (0.79)	3.05 (0.95)	3.35 (1.18)	1.855	0.165	–
T2	2.45 (0.89)	2.82 (1.14)	3.30 (1.08)	3.329	0.043	SPICE-users > Non-users
Diff. T2-T0	−0.55 (0.76)	−0.45 (1.06)	−0.35 (0.93)	0.232	0.794	–
*p*	0.008	0.053	0.100			
ASI – Heightened Emotionality (HE)						
T0	2.85 (1.18)	3.00 (1.41)	3.85 (1.09)	3.801	0.028	SPICE-users > Non-users
T1	2.60 (1.10)	2.77 (1.31)	3.35 (1.04)	2.311	0.108	–
T2	2.20 (0.95)	2.73 (1.20)	3.25 (0.91)	5.137	0.009	SPICE-users > Non-users
Diff. T2-T0	−0.65 (0.75)	−0.27 (0.83)	−0.60 (0.60)	1.662	0.199	–
*p*	0.004	0.130	0.001			
ASI – Heightened Cognition (HC)						
T0	3.00 (1.12)	3.32 (1.13)	3.60 (1.23)	1.336	0.271	–
T1	2.85 (0.99)	3.14 (1.08)	3.70 (1.03)	3.492	0.037	SPICE-users > Non-users
T2	2.35 (0.99)	2.91 (1.19)	3.50 (1.00)	5.793	0.005	SPICE-users > Non-users
Diff. T2-T0	−0.65 (0.81)	−0.41 (0.59)	−0.10 (0.97)	2.382	0.101	–
*p*	0.003	0.007	0.713			

Data are reported as mean (SD). Statistics: one-way ANOVA, Tukey post-hoc test and Wilcoxon test. ASI: aberrant salience inventory.

The overall PANSS score globally reduced from T0 to T2 within all three groups. Regarding the PANSS positive subscale, post-hoc analysis between groups showed significantly higher values for SPICE-users compared to both THC-users and non-users. This difference persisted overall observation times (T0: *F* = 5.218, *p* = 0.008; T1: *F* = 7.982, *p* = 0.001; T2: *F* = 18.367, *p* < 0.000). Moreover, positive PANSS scores in SPICE users registered a narrower reduction after treatment compared to the other two groups. Conversely, the negative subscale of PANSS showed higher scores in non-user groups compared to THC-users and SPICE-users in all observations (T0: *F* = 13.869; T1: *F* = 17.163; T2: *F* = 16.048; *p* < 0.000).

A global improvement in overall functioning was observed throughout the entire observation period. At T2, GAF scores of non-users exhibited a trend toward significance, indicating higher values compared to SPICE-users (*F* = 4.901, *p* = 0.051). Additionally, non-users demonstrated greater improvements in GAF scores from T0 to T2 compared to SPICE users.

DES-II scores showed significantly higher values for both THC-users and SPICE-users over non-users during the entire observation time (T0: *F* = 9.414; T1: *F* = 12.968; T2: *F* = 14.625; *p* < 0.000). No statistical differences between THC-users and SPICE-users have been detected. THC users’ score improvement from T0 to T2 is significantly lower than non-users.

SSI score resulted higher in SPICE-users compared to both THC-users and non-users in all three observations (Table 2).

### 3.3 Correlation between aberrant salience and psychosis onset in non-users, THC-users and SPICE-users

To assess psychometric scores of SA between and within groups, one-way ANOVA, Tukey post-hoc, and Wilcoxon tests were performed. All groups manifested a reduction in global and subscale ASI scores between T0 and T2. The ASI total score was greater in SPICE-users than in non-users in all three observations, while the improvement in the global score between T2 and T0 was smaller in SPICE users compared to non-users. No significant differences were found between THC-users and other groups. Within the subscale *FIS*, no relevant differences were found among groups at different observations: all registered a decrease at T2, but only at T1 SPICE-users had significant greater values on THC-users. Within the subscale *SS*, SPICE-users reported greater values in all observations (T0: *F* = 16.589; T1: *F* = 18.346; T2: *F* = 23.289; *p* < 0.000) compared to the other two groups. In subscale *IU*, SPICE-users manifested higher scores compared to non-users, but only at T2 it was significant (*F* = 3.329, *p* = 0.043). Within *HE* subscale, SPICE-users manifested greater scores than non-users, significant both at T0 (*F* = 3.901 *p* = 0.028) and T2 (*F* = 5.137 *p* = 0.009). For *HC* subscale, SPICE-users manifested higher values than non-users, but only at T1 (*F* = 3.492 *p* = 0.037) and T2 (*F* = 5.793 *p* = 0.005) differences were significative. See Table 3.

Spearman’s correlation was performed to investigate relations among psychometric and ASI scales. The results are reported in Table 4. After Bonferroni correction for multiple comparisons (0.005/14 = 0.004), a negative correlation has been reported between the negative PANSS score and the SS subscale (Spearman’s rho coefficient = −0.463; *p* < 0.000). See Table 4.

**Table 4:** Correlations in the whole sample.

		ASI total	ASI – FIS	ASI – SS	ASI – IU	ASI – HE	ASI – HC
PANSS positive	Rho	0.244	0.167	0.176	0.142	0.253^*^	0.120
	*p*	0.056	0.195	0.172	0.272	0.047	0.352
PANSS negative	rho	−0.165	−0.031	−0.463^**^	−0.230	−0.007	0.080
	*p*	0.199	0.810	0.000	0.072	0.958	0.537
PANSS general	rho	0.027	−0.249	0.134	−0.050	0.079	0.116
	*p*	0.837	0.051	0.299	0.701	0.543	0.370
GAF	rho	−0.109	−0.146	0.082	−0.004	−0.115	−0.131
	*p*	0.400	0.257	0.527	0.974	0.373	0.310
DES-2	rho	0.207	−0.014	0.292^*^	0.202	0.297^*^	0.131
	*p*	0.106	0.912	0.021	0.115	0.019	0.309
SSI	rho	0.192	0.178	0.317^*^	0.071	0.097	0.194
	*p*	0.136	0.166	0.012	0.584	0.455	0.131
Sex	rho	0.099	0.139	−0.011	0.111	0.172	0.048
	*p*	0.445	0.280	0.931	0.389	0.182	0.713
Age	rho	−0.243	−0.212	−0.367^**^	−0.334^**^	−0.149	0.084
	*p*	0.057	0.097	0.003	0.008	0.249	0.517

Statistics: Spearman’s rho coefficient. Bonferroni correction for multiple comparison: 0.05/14 = 0.004.

## 4 Discussion

This paper presents an effort to compare three groups: non-users of cannabis, THC-users, and SPICE-users. The comparison is conducted using the PANSS, the SSI, the GAF scale and the DES II. These assessments were carried out within each individual group and across all three groups, both at the onset of psychosis and during the follow-up period with antipsychotic treatment in real-life scenarios. One of the key innovations in this study is the focused assessment of the role of AS in the onset and progression of psychosis within the three groups of patients. Additionally, the study explores how the specific subscales of the ASI contribute to the development and course of symptoms within each group and when comparing the three groups. Furthermore, the study offers a critical comparison between ASI and its subscales and other psychopathological dimensions such as psychotic symptoms, suicidality, global functioning, and dissociation.

### 4.1 Psychotic symptoms

The overall PANSS scores showed a reduction in all three groups at the end of the observation and treatment period. However, there were notable differences in the improvement of positive symptoms. SPICE-users exhibited more severe positive symptoms (25, 27, 39) compared to both NC users and non-users, in line with existing literature (26, 60–62). Importantly, during the final evaluation, SPICE-users showed a less pronounced reduction in positive symptoms compared to the other two groups, confirming our previous research (21). This suggests that SCs may trigger more severe and persistent positive symptoms with a poor response to antipsychotic treatments (9, 63–65). In fact, the majority of SCs are more potent than THC, functioning as full agonists at the CB1 receptor (9). SCs typically exhibit a 4 to 5 times higher affinity and a 40 to 60 times higher potency compared to THC (65). Furthermore, the mechanisms responsible for the potential of NC and, notably, SCs to induce psychotic symptoms appear to be linked to the functioning of the dopamine, GABA, and glutamatergic systems (11, 12).

Regarding negative psychotic symptoms, they were more pronounced in non-users compared to both THC-users and SPICE-users, consistent with prior literature (23, 26, 32, 33) and our previous study (21). Two hypotheses could explain these findings. First, the more persistent positive symptoms might overshadow the negative ones, becoming the dominant clinical manifestations. Second, SCs may induce more significant dopaminergic activation compared to NC, leading to the emergence of more prominent positive symptoms (64).

### 4.2 Global functioning

Regarding global functioning, there was initially a comparable improvement across all three groups, but after the final evaluation, GAF scores indicated a trend of higher values in SPICE-users, suggesting that non-users had a better recovery. This aligns with findings in the literature (21, 27, 32, 39) and our recent study (21). This observation may imply that SCs have a more adverse impact on global functioning, even after the clinical course resolves. Additionally, it’s worth considering the hypothesis that individuals with a history of poorer global functioning may be more inclined to use potent substances like SCs.

### 4.3 Dissociative symptoms

Dissociative symptoms in the three samples were significantly greater in substance users than in non-users, in line with previous studies (26, 55) during the last evaluation the group in which such symptoms remained significantly less reduced was the THC-users. This data could provide additional support for the notion that NC has a greater potential to induce dissociation, irrespective of the presence of positive symptoms (21).

### 4.4 Suicidal ideation

Suicidal ideation was consistently more prevalent among SPICE-users in each assessment, compared to both THC-users and non-users. This heightened suicidal ideation in SPICE-users may be indirectly related to the increased impulsivity induced by SCs and the persistence of positive symptoms along with compromised overall functioning (21, 55, 61, 62, 66). The role of suicidality is particularly significant in the context of substance use disorders, especially concerning NPS that share some pharmacodynamic properties with SCs (6, 67).

### 4.5 Aberrant salience

The neurobiological concept of AS is based on the notion introduced by Conrad (68) as “Trema,” involving the sensation that something of great importance is imminent. This sensation transforms the perceptual background, making everything in the periphery or not part of the current focus potentially threatening, with the dominant quality of the delusion spreading across the entire perceptual field. All three groups showed a decrease in both overall and subscale scores on the ASI throughout the entire observation period. Notably, SPICE-users consistently had higher ASI scores compared to non-users, and they exhibited a less pronounced response to therapy across all three assessments. This novel observation highlights an increased AS in SPICE-users and a limited reduction of AS in these patients following psychopharmacotherapy. One hypothesis that could be considered is that this data may be linked to the heightened dopaminergic activation induced by SPICE. Mesolimbic dopaminergic hyperfunction has been proposed to drive maladaptive associative learning early in the course of the disease (69, 70), with the limited efficacy of antipsychotics, which act via blockade of the D2 receptor, correlating with insufficient targeting to reverse aberrant salience processing (69).

However, no significant differences were found between THC-users and the other groups, despite some prior literature suggesting increased AS in generic THC-users. Some studies have indicated that THC administration can lead to abnormal salience processing in healthy individuals, resembling patterns seen in individuals with psychosis. However, the precise impact and mechanisms by which cannabis modulates AS remain unclear and require further investigation (71). It’s worth noting that AS is positively correlated with the severity of cannabis-induced psychotic symptoms but is not consistently observed in all cannabis-users. This suggests that the connection between cannabis use and psychosis may involve disruptions in salience processing (72).

#### 4.5.1 Feelings of increased significance

FIS represents heightened attribution of importance to stimuli that would typically be considered insignificant (47, 73). While all three groups showed a reduction in this factor following antipsychotic therapy, it’s crucial to note that during the initial observation SPICE-users had higher scores compared to NC-users. This finding supports the hypothesis that SCs may induce a more pronounced dysregulation of dopaminergic and opioidergic systems, potentially contributing to this phenomenon.

#### 4.5.2 Sense sharpening

SS refers to anomalies of perception characterized by a subjective sensation of heightened sensory acuity that makes previously insignificant stimuli noticeable. It appears to relate to prepsychotic experiences and may have broader relevance to the prodromal stage of schizophrenia than the feeling of increased significance alone (47). This concept aligns with our findings, as it was more prevalent than the feeling of increased significance in individuals with psychosis who used SPICE across all observations and did not improve after therapy. This could be linked to the greater severity and persistence of positive symptoms in this group of patients.

#### 4.5.3 Impending understanding

IU this factor suggests that individuals experiencing AS may perceive these heightened feelings of significance as leading to a breakthrough in understanding (47). SPICE-users displayed higher scores in this factor compared to non-users, and notably, only non-users showed a response to therapy. This data could potentially be attributed to both the heightened severity and persistence of positive symptoms and the poorer overall functioning observed in SPICE-users.

#### 4.5.4 Heightened emotionality and heightened cognition

HE and HC may result from a person attempting to understand the emotions and cognitions that accompany an AS experience but may also be more general to pre-psychotic experiences (47). This state creates anxiety and confusion between the perception of oneself and that of the external world (74). In both factors, SPICE-users exhibited higher scores than non-users, and there was a significant deterioration of symptoms among SPICE-users compared to non-users at the end of treatment. We can hypothesize that the use of SCs, characterized by somatic symptoms, anxiety, agitation, cognitive impairment, suicidal ideation (34), catatonia, depersonalization, dissociation, greater positive symptoms, and fewer negative symptoms (23, 32, 33) could lead to greater confusion and perplexity in the perception of themselves and the world around them.

Lastly, it’s important to note a negative correlation between negative symptoms and the SS subscale. As previously discussed, this association could be attributed not only to the neurobiological effects of SPICE but also to the heightened severity and persistence of positive symptoms observed in this group of patients.

Another research perspective involves the role of AS in the reward mechanism. This view, called the “*incentive salience theory of addiction*,” predicts that repeated exposure to addictive substances can lead to persistent changes in neuronal pathways involved in the attribution of salience to stimuli. At the behavioral level, incentive salience produces interference in cognitive processes by shifting attention to the substance itself (*compulsive wanting*). Thus, the increased AS detected in SPICE patients may justify the heavy craving for the substance (75, 76).

## 5 Limitation

Several limitations can be reported in our study: (i) the study included a low number of participants for a disorder that is very heterogeneous in its clinical manifestations; (ii) a 6 month period may not be sufficient to draw conclusions regarding clinical outcomes; (iii) the pharmacological treatment included a wide variety of antipsychotic treatments; (iv) knowledge of which types of substances were used was obtained through clinical interviews with patients and family members; (v) finally, the ASI questionnaire, used to evaluate the AS experience, is a self-report measure; hence, it might have been under- or over-estimated, or possibly misunderstood.

## 6 Conclusion

Despite some limitations and the need for further research, our study lays the groundwork for a preliminary understanding of the distinct mechanisms of action of THC and SPICE in triggering psychotic disorders with specific symptomatic characteristics during the early stages and over a longitudinal course. Notably, during the final evaluation at 6 months, individuals using SPICE exhibited more prominent and persistent positive symptoms, a worse recovery in global functioning and a poorer response to therapy in contrast to both THC-users and those who refrained from cannabis use. Conversely, negative symptoms were primarily observed among non-users. Significantly, our study aims to provide the fundamental basis for unraveling the connection between AS and psychosis, whether it is induced by substance use or not. This effort is notably evident in the elevated overall ASI scores observed among SPICE-users, particularly within the SS subscale. Additionally, their limited improvement in AS over the entire observation period is noteworthy. These insights hold potential implications for the identification of specific pharmacological interventions tailored to address these mechanisms.

## Data availability statement

The raw data supporting the conclusions of this article will be made available by the authors, without undue reservation.

## Ethics statement

The studies involving humans were approved by SS. Annunziata Hospital – University G. d’Annunzio Ethical Committee (reference code: CHPN189, 26 January 2012). The studies were conducted in accordance with the local legislation and institutional requirements. Written informed consent for participation in this study was provided by the participants’ legal guardians/next of kin.

## Author contributions

VR: Conceptualization, Methodology, Writing – original draft, Data curation, Investigation, Writing – review & editing. IM: Conceptualization, Writing – review & editing. FC: Conceptualization, Data curation, Formal analysis, Writing – review & editing. FDC: Data curation, Formal analysis, Methodology, Writing – review & editing. GlM: Conceptualization, Investigation, Data curation, Writing – review & editing. TP: Data curation, Methodology, Writing – review & editing. AP: Conceptualization, Methodology, Writing – review & editing. MP: Conceptualization, Investigation, Writing – review & editing. FV: Conceptualization, Data curation, Writing – review & editing. DB: Conceptualization, Supervision, Writing – review & editing. GvM: Conceptualization, Investigation, Methodology, Writing – review & editing. GsM: Conceptualization, Data curation, Methodology, Writing – review & editing.
