# Supplementary material for: Aberrant salience in cannabis-induced psychosis: a comparative study
Source: Front Psychiatry. 2024 Jan 8;14:1343884. doi: 10.3389/fpsyt.2023.1343884 (PMC10801803; doi:10.3389/fpsyt.2023.1343884)
Supplement: Supplementary file 1 [file Data_Sheet_1.docx]

**Factor 1: Increased Significance.**

1. Do certain trivial things ever suddenly seem especially important or significant to you?

5. Do you sometimes notice small details that you have not noticed before that seem

important?

10. Do you ever feel the need to make sense of seemingly random situations or occurrences?

16. Do you sometimes attribute importance to objects which you normally would not?

21. Do you often become fascinated by the little things around you?

27. Have you sometimes become interested in people, events, places, or ideas that normally

would not make an impression on you?

15. Do you go through periods in which songs sometimes seem to have an important

meaning for your life?

**Factor 2: Senses Sharpening.**

22. Do your senses ever seem extremely strong or clear?

3. Do your senses sometimes seem sharpened?

12. Do you sometimes feel that you can hear with a greater clarity?

18. Has your sense of taste ever seemed more acute?

9. Do you ever go through periods of heightened awareness?

**Factor 3: Impending Understanding**

2. Do you sometimes feel like you are on the verge of something really big, but you’re not

sure, what it is?

6. Do you sometimes feel like it is important for you to figure something out, but you’re

not sure what it is?

11. Do you sometimes feel like you are finding the missing piece to a puzzle?

17. Do you sometimes feel like you are on the verge of figuring out something really big or

important, but you aren’t sure what it is?

29. Do you sometimes notice things that you haven’t noticed before that take on a special

significance?

**Factor 4: Heightened Emotionality.**

8. Do you ever have difficulty telling if you are thrilled, frightened, pained, or anxious?

14. Do normally trivial observations sometimes take on an ominous significance?

20. Do you go through periods in which you feel overstimulated by things or experiences

that are normally manageable?

24. Do you ever feel that your boundaries between inner and outer sensations have been

removed?

26. Do you ever have a feeling of inexpressible urgency, and you are not sure what to do?

28. Do your thoughts and perceptions ever come faster than can be assimilated?

**Factor 5: Heightened Cognition**.

4. Do you ever feel like you are rapidly approaching the height of your intellectual powers?

25. Do you sometimes feel like the world is changing and you are searching for an

explanation?

7. Do you ever go through periods where you feel especially religious or mystical?

13. Do you sometimes feel like you are an especially spiritually evolved person?

19. Do you ever feel like the mysteries of the universe are revealing themselves to you?

23. Do you ever feel like a whole world is opening up to you?
